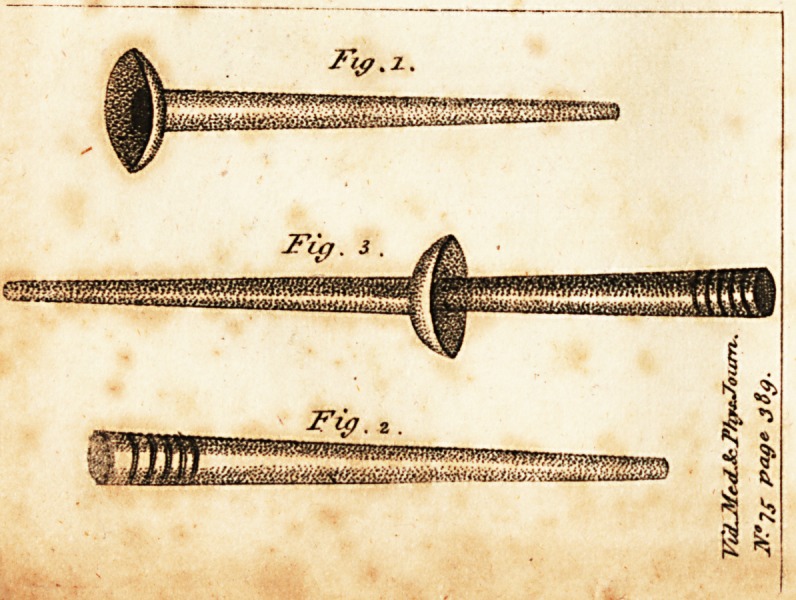# Observations on Select Subjects in Surgery

**Published:** 1805-05-01

**Authors:** 


### Medical and Phyfical Journal.

VOL. XIII.] MAY 1, 1805. [NO. LXXV.

*Printed for R. PHILLIPS, by W. Thorne, Red Lion Court, Fleet Street, London.*

### OBSERVATIONS ON SELECT SUBJECTS IN SURGERY. BY MR. SIMMONS.

( Continued from pp. 289—294. )

### PULMONARY OSSIFICATIONS.

OSSIFICATIONS, or bony concretions, out of the usual course of nature, are most frequently met with in the investing membranes lining cavities, such as the pleura and dura mater; and in the arterial system, as in the aorta, and its various ramifications. The elements which constitute bone may be deposited in any other part of the body, though less frequently found so; and I am now about to record some specimens, in which the deposition had taken place in the lungs. These osseous concretions were found at the root of the lungs, on each side of the chest, and I apprehend, had not much impeded respiration, as in other respects there was no appearance of disease. But I am not acquainted with the previous history of the case.

### TENDINOUS OSSIFICATIONS.

Concretions of bone in the voluntary muscles are, I believe, seldom met with. Two or three instances only have fallen under my observation; and in these the bone had formed in the tendinous expansion of the biceps flexor cubiti. In the one, of which a representation is here given of the ossification, the motions of the left arm were so impeded by it, and attended with so much pain, that the young man requested me to take it out for him, which I did accordingly. The wound healed without any trouble, and he had afterwards the full use of his arm.

### ON THE GENERAL TREATMENT OF CONTUSION.

It is hardly necessary to define what is meant by a contusion, or, in common language, a bruise, it is in general so well understood; nor is it less generally known, that it is occasioned by force, or external violence. Its effects however are greatly diversified, and depend not only on the peculiar structure of the part injured, but also on the degree of violence exerted in inflicting the injury. When the shock is general, with or without any particular organic lesion, the vital powers are so depressed as, in some instances, to threaten a temporary suspension of them; yet in proportion to the magnitude of the effect on the system, is the necessity for the withdrawing of blood very generally rated. But, I have long considered the practice founded on this opinion as at least erroneous; and under this persuasion, instead of employing the lancet, I apply warmth to the extremities, and let the patient take of warm and transient stimulants, in order to promote perspiration without inflaming the habit. If the reaction afterwards should be considerable, then I order phlebotomy, fully and freely, as the symptoms may indicate; and exhibit saline purgatives in doses adequate to produce copious evacuations. But, in the febrile state, instead of saline mixture, or preparations of antimony, I have for some years substituted the digitalis, in half-grain doses, along with an equal quantity of opium, every six hours. The well-known properties of the digitalis of acting upon the absorbents, and lowering the pulse, point it out as a remedy well calculated to promote the absorption of the extravasated fluids in contusion, as well as to prevent the necessity of profuse and excessive bleeding, and in both these intentions my wishes have been fully realized by it.

### Excoriation, and Ulcers on the Glans and Prepuce.

Some men are subject to a discharge from the glans and prepuce, which at times is very troublesome to them, and not only productive of extensive excoriations, but not unfrequently these degenerate in parts into small ulcers. Those who have an irritable skin, and in whom the prepuce is somewhat contracted, are most susceptible of these affections. In general, a vitriolic, or other astringent wash, will very soon suppress this increased secretion, and heal the ulcers; and the recurrence of both may be prevented by ablution with soap and water two or three times a week. When the ulcers are intractable to such applications, it will become needful to destroy their surface with the nitrated silver, or vitriol of copper. No internal medicine dicine is required, as the disease is entirely local, and has nothing of syphilis in it. In one case only I was baffled for some time; in this the secretion was suppressed by the means above recommended, but the ulcers, several in number, although their surface was repeatedly destroyed, were successively covered with a whitish coloured coat, and would not heal. I at length discovered, that the patient, in his anxiety, was very frequently looking at and washing the ulcers with warm water; and, upon the supposition that their obstinacy proceeded from this cause, the crust acting as a defence to the tender surface underneath, I advised him to desist from such frequent dressings, and not to expose them oftener than once a day; and, upon following this advice, they were all cicatrized in a few days.

The female organs are obnoxious to a similar affection, which, in these, is curable by the like means.

### On White Gangrene

Soon after my attendance on the home patients commenced at the Manchester Infirmary, &c. in the year 1790, I was called to see two cases of gangrene, in which the sloughs had assumed an opake whiteness, instead of the dark hue of gangrenous exfoliations. In both these instances the patients were females, who were in the last stage of dropsical debility; and in each the slough had formed upon the upper side of the foot, just below the instep. It might be said, with almost literal veracity, that both these patients were exanguious, so pallid were they, and destitute of invigorated action. It is hardly necessary to observe, that both these women died; nor can a recovery under such circumstances be rationally hoped for. To produce such appearances of extreme feebleness, life, gradually wasted, must be on the eve of extinction, and render all means of support ineffectual.

### On an Enlargement of the Uterus.

Owing to an accident, I am prevented from supplying a drawing of this singular instance of enlargement of the uterus. Nevertheless, a verbal description of it may not be entirely uninteresting, though not attended with any practical utility. With the early history of the case I am unacquainted, having seen the patient a short time only before her decease. But, after death, I, with some difficulty, possessed myself of the uterus, which was enlarged to a very considerable magnitude, for it weighed twenty-four

 pounds pounds avoirdupois. The diseased alteration consisted not in any scirrhosity, or cancerous induration, but in an enlargement or distention of the uterus, produced by the deposition of carnous layers within its cavity, with here and there membranous intersections that formed bags, which were filled with a limpid fluid. Both the ovaria were sound, as well as the round and broad ligaments. The neck of the uterus had disappeared, as is customary in advanced pregnancy; but the vagina and os uteri were smooth, and free from any morbid appearance whatever. With very little force, I passed a long silver probe through the os tincæ, between the inner surface of the uterus and the layer contiguous to it, down to the fundus uteri; which pretty clearly indicated, that the layer was a deposition merely, and had not firmly agglutinated. And this opinion is further corroborated by the not very irregular flowing of the catamenia (as she herself informed me) till it ceased spontaneously only a few years before her death. The appearances in the abdomen were those of chronic peritoneal inflammation; several of the viscera were coated over with layers of coagulable lymph, that had assumed an almost membranous consistence; and there was also a quantity of a wheyish coloured fluid, interspersed with lumps, and broken membranous coats of lymph coagulated. The pressure of this enormous mass had likewise occasioned a contraction in the upper portion of the rectum, so that at times, she was troubled with obstinate costiveness.

### On the Adipose Sarcoma.

The opinion that the Adipose Sarcoma originates in the deposition of coagulable lymph, may perhaps admit of some exceptions. One is, that the adipose cells themselves may, to a limited extent, receive such a morbid impression, as to lead to the formation of sarcoma, which would thence progressively augment in bulk.

H. A. ætat. 36, had a sarcoma about the size of an orange, situated above, and posterior to the hip, on her right side, the growth of which, as it was then of two years standing, had been slow, and unattended with pain. On the 4th of February, I removed it by incision; and on examination after its removal, one lobule, of equal magnitude with the rest, evidently consisted in an enlargement of an adipose cell, whose colour and consistency were scarcely altered, though the other lobules, apparently of the same origin, had acquired the customary degree of density.

Contiguous

Contiguous to the tumour, the adipose substance was abundant, and in parts indurated, so as to require the extirpation of a considerable portion of it also; but whether the induration was the effect of disease or of pressure, I am unable to say.

### On the Hydrocele.

The term Hydrocele is technically restricted to a collection of watery fluid in the tunica vaginalis of the scrotum, although it might designate the accumulation of a limpid fluid in any other part of the body with equal propriety.

In the treatment of this complaint it has been proposed to relieve the present incumbrance by what has been called the *palliative* cure, which consists in puncturing the sac with a small trocar, and letting out the water. For obtaining the second or *radical* cure, surgeons have recommended many different methods, the principal of which are, 1. Incision; 2. Excision of the cyst; 3. Caustic; 4. The tent; 5. The seton; 6. Injection. But as the latter gives comparatively little pain, and excites only a very manageable degree of inflammation, it is now very generally preferred in practice. To facilitate the execution of it, therefore, I have invented a small silver tube, the taper end of which is made to fit accurately into the canula of the trocar; and the other extremity, of a larger diameter, is marked with several circular grooves like a glyster-pipe. To this extremity a bladder, to contain the stimulating fluid for injection, may be appended, in the manner of arming a glyster-pipe with a bag; and, if a bladder of a proper size be chosen, whatever may be the dimensions of the cavity, it may be fully distended by the fluid thus expressed.

The spontaneous cure of a hydrocele is not a very common occurrence; I once met with an instance of it in an elderly man, who had carried the disease for several years. At length the distention became excessive, and similar to what takes place in an over distension of the urinary bladder; a circular slough formed about the bigness of a shilling, near the middle of the tumour on the anterior surface. On the exfoliation of the slough, the general cavity was exposed, and inflamed throughout so completely, as to prevent any re-accumulation of fluid.

I saw this man several years afterwards, when he had suffered no return of the complaint.

Explanation

### Explanation of the Plates.

Plate 1.—In this plate the upper figure represents the small hook, recommended for the net-retractor in amputation; and the lower one the file; the back of which should be somewhat broader than is here delineated.

Plate 2.—Is a delineation of the steatoma, in which will be observed the lobulated appearance of that species of tumour, and the lobe, with a narrow neck, that was descending towards the left clavicle. The portion of skin that was left adhering, is also distinctly marked.

Plate 3.—In plate the third is represented the scrofulous testis, and diseased portion of omentum. In the upper figure is seen the morbid appearance assumed by the body of the testis after its exposure, by a longitudinal section. The cord at the lower extremity is the vas deferens, which was inserted into the cleft between the two hemispheres.

In the lower figure, the tuberculated border is the part of the omentum that was diseased.

Plate 4.—Is represented the alteration of structure produced in the testicle by scirrhus, before any degree of ulceration had taken place, or the spermatic cord had enlarged. The appearances are very accurately given.

Plate 5.—In plate five, the morbid appearances exhibited by cancer in the ulcerated stage are delineated. The drawing was made from the breast of a married woman, not very young, who had borne many children. The ulcerated parts around the nipple are duly characterized, as well as the scirrhous knots to the left, which are common in the advanced stage of cancer.

Plate 6.—In plate six, O. L. represents some specimens of pulmonary ossification; and O. E. a longitudinal section of one of these; the moiety to the left representing the inner, and the other to the right, the outer structure of an ossification of larger dimensions. O. T. exhibits the ossification taken out of the tendinous expansion of the biceps flexor cubiti.

Plate 7. Fig. 1 in the engraving, refers to the silver tube. Fig. 2, to the canula of the trocar. Fig. 3, to the canula and tube in conjunction. They are all represented of the natural size.

If any utility should acrue from the publication of these drawings, the public will feel its obligations to the ingenious Mr. Patrick Mac Morland, by whom I have been favoured with them. They are faithful representations of Nature, and are executed in a style of peculiar neatness.

## Figures and Tables

**Fig. 1. Fig. 2. Fig. 3 f1:**